# Impact of vision problems on children’s daily activities: Insights from a focus group discussion

**DOI:** 10.12688/f1000research.159464.1

**Published:** 2024-12-20

**Authors:** Tshubelela Sello Simon Magakwe, Rekha Hansraj, Zamadonda Nokuthula Xulu-Kasaba

**Affiliations:** 1 Discipline of Optometry, University of KwaZulu Natal, Durban, Durban, 4000, South Africa

**Keywords:** Focus group discussion, children's vision, visual impairment, quality of life, poor vision, rural school-going children, South Africa.

## Abstract

**Background/Purpose:**

There are approximately 18.94 million visually impaired children worldwide, with 90% of them living in low-middle income countries. Research shows that visual impairment (VI) has a significant impact on the quality of life (QoL) of those affected. Therefore, it is essential to investigate how VI affects the daily activities of these children to develop management strategies that can help minimize its impact on their QoL. Therefore, qualitative research design was utilized to explore the perceived impact of VI on the daily activities of children living with VI.

**Methods:**

Using a qualitative approach, purposive sampling was used to identify information-rich participants to partake in focus group discussions (FGDs) and address the aim of the study adequately. Identified data sources were divided into two groups: one with ‘experts’ who worked with children regularly, and another with school-going children aged 6-17 years, from three rural schools in Sekhukhune district, South Africa. The FGDs were guided by semi-structured interview questions. All sessions were recorded, transcribed verbatim, cleaned, coded, and analysed under ten domains, identified from the literature.

**Results:**

A total of 477 statements and comments related to children’s vision were generated through the nine FGDs. Participants were nine experts working with children, and 49 children living with or without VI. Of the total number of statements, 60% (n = 287) were generated from FGDs with children, 63% (n = 299) were negative statements, and the remaining 19% (n = 92), and 18% (n = 86) were neutral and positive statements respectively. The most generated statements fell under the domains ‘Hobbies, Leisure and Sport’ 21% (n = 101), ‘Education’ 21% (n = 100), and ‘Psychological and Emotional’ 12.8% (n = 61).

**Conclusion:**

This study offered comprehensive insights into the impact of VI on the daily activities of rural school-aged children and young people.

## Introduction

Visual impairment (VI) is the most common cause of childhood handicaps and the second leading cause of childhood blindness. ^1, 2^ It limits the ability of children to perform well in school, as a major part of learning depends on vision. ^3^ Additionally, children with VI often struggle with social and recreational activities, with subsequent negative effects on their socioeconomic security, all impacting their quality of life. ^3– 5^ The onset of VI before or during school-going age has a significant impact compared to that in adulthood, considering the greater number of years that they will likely carry this condition. ^6^ In the United States, it was reported that a person living with VI needs an estimated $4000 per annum for medical expenditure, an amount that increases with age, as it later includes the cost imposed by loss of productivity, anxiety, and frustrations linked to their impairment. ^7, 8^

Globally, there are around 18.94 million visually impaired children, with 1.42 million of these children being blind. ^9^ The leading cause of VI in children is uncorrected or inappropriately corrected refractive error, attributing to about 12.8 million cases. ^5^ Ninety percent of children with VI reside in low-to-middle income countries (LMICs). ^10, 11^ It is essential to understand the impact of VI on children’s daily lives and their perceptions about their abilities and difficulties with daily activities to facilitate the development of appropriate management strategies.

To explore this, FGDs were decided upon for this study as a standard method for exploring people’s feelings, motivations, insights, and experiences on any topic. ^12, 13^ Focus group discussions are regarded as a quick and cost-effective qualitative research method in which participants openly share their perceptions and jointly develop new insights and a deeper understanding of the issues in question. ^12, 13^ The purpose of this particular focus group study was to identify activities that school-aged children from rural areas in South Africa engage in, the difficulties that children with VI can encounter with these activities, and how their lives are impacted as a result of this limitation. The ultimate goal is to develop a self-reported measure of visual function QoL for rural school-going children aged 6-17 years.

Numerous instruments are available to measure the vision-related quality of life (QoL) in adults, which have been globally developed and validated with good reliability. However, there has been insufficient effort in creating similar instruments for school-aged children. ^14^ In most cases, the tools designed for adults need to be adapted before being used on children. This adaptation process may compromise the established reliability and validity. The WHOQOL group has suggested that each country should create its own instrument to measure the impact of any disease and treatment, which should be sensitive to the culture and suitable for different age groups. ^15^ A recent review of instruments for assessing vision-related quality of life in children and adolescents revealed a lack of such instruments for children in LMICs. Therefore, it is essential to explore these children’s perceptions and statements to guide the development of an appropriate tool that will allow learners to honestly evaluate themselves, rather than being evaluated solely based on test results from eye care practitioners.

## Methods

This qualitative study’s aim was to gather comments and statements about the impact of VI on children’s daily lives. FGDs were used to collect data for the study. The VI was classified based on the visual acuity (VA) measurement as guided by the World Health Organization (WHO). ^16^ Mild VI was defined as having a presenting VA in the better eye of less than 0.30 logarithm of the minimum angle of resolution (LogMAR) but greater than or equal to 0.50 LogMAR. Moderate VI is defined as having a presenting VA in the better eye of less than 0.50 LogMAR but greater than or equal to 1.0 LogMAR. Severe VI was defined as having a presenting VA in the better eye of less than 1.0 LogMAR but greater than or equal to 1.30 LogMAR. Blindness was defined as having a presenting VA in the better eye of less than 1.30 LogMAR. ^16^

The research followed the principles of the Declaration of Helsinki regarding research involving humans, and ethical clearance was obtained from the Biomedical Research Ethical Committee of the University of KwaZulu-Natal (BREC/00003939/2022). Consent and assent forms were provided and signed by experts, children, and their parents prior to data collection. A total of nine FGDs were conducted.

### Focus group discussions with experts

In the course of two FGDs, a total of nine professionals with an interest in children or working with children, participated. A purposive sampling method was used to recruit the participants, which included one nurse, two occupational therapists, two optometrists, and four teachers from both mainstream and special schools.

The FGDs were conducted online and recorded on Microsoft Teams to accommodate the participants’ preferences and scheduling constraints. Prior to the discussions, participants each received an information sheet about the study, and an invitation to participate. After confirming voluntary consent, they received a Microsoft Teams meeting invitation via their personal or work emails, detailing the date and time of the meeting. The principal investigator (PI) facilitated the discussion with an assistant by a trained moderator who took notes. Participants were welcomed, introduced to the PI and moderator, and given an overview of the purpose and topic of the discussion. They were also informed that there were no right or wrong answers, only differing points of view, and that the meeting would be recorded, so one person would speak at a time. The discussions were guided by prompt questions related to vision and children’s activities as detailed below:

1. What role does vision play in children’s lives in general?
2. What activities, at school, require children to have good vision?
3. What types of recreational activities that children engage in require good eyesight?
4. Does a child’s vision impact the manner in which they socialise?
5. If a child has vision problems, how would this impact his/her life and development?

Each meeting lasted between 75 and 90 minutes and was audio recorded. At the end of each discussion, the PI and moderator thanked the participants for their time and contributions. They then stayed for a few minutes to summarize important points and emotions captured during the discussion.

### Focus group discussion with children

Forty-nine children aged 6 to 17 years from three rural schools in Sekhukhune District, Makhuduthamaga Municipality, Limpopo province, were involved in one of seven FGDs. They were all of African ethnicity and were selected from Bosele Special School, Jane Furse Comprehensive, and Moreko Secondary School. The FGDs were divided into two sessions per school, one for the age group 6–11-year-olds and another for the 12–17-year-olds. Each group comprised solely of the children from one school but divided according to the two age groups, hence six groups in total. A seventh FGD was conducted for children from Bosele Special School in the age group 6-11 years after the initial round of FGDs, due to the need for more data. The groups were also stratified based on gender and visual status.

The FGDs took place at the respective school premises in an allocated classroom. Vision screening tests, to obtain clinical data for another part of the broader study, were conducted a few days prior to the discussions, and these included visual acuity testing, fundus examination, and auto refraction. All participating children were proficient in English and did not have any impairments apart from vision. The discussions took place in the afternoon to avoid disruption of academic activities. On the day of the FGD, the children were introduced informally over a light lunch before the commencement of the discussion. The discussions, led by the PI with a moderator was taking notes, each lasted between 60-80 minutes and was audio-recorded. Children were seated during the group discussions. After a set of ground rules were established, discussions commenced, structured as open-ended questions, and further expanded using probing questions whenever necessary. At the end, participants were acknowledged for their valued contribution and time. Both the PI and the moderator remained for a summary and to conclude the discussion.

### Data capturing and analysis

The recordings and the transcription were done via Microsoft Teams meeting, and then PI and SS listened to the recording several times to verify transcriptions, clean data and extract important information from the interviews. This study employed Template Analysis as a method for thematic analysis to gather, categorize, and analyse data. ^17^ Template Analysis is a flexible approach that provides a structured way to organise and analyse large amounts of rich qualitative data, addressing common challenges in qualitative research. ^18, 19^ This technique allows for the use of a priori themes and iterative refinement of the coding template, making it suitable for diverse research contexts as in the current study, and was conducted via six steps as outlined below. ^17, 18^

Step 1: To familiarize ourselves with the data to be analysed, two individuals read the material several times.

Step 2: The preliminary coding step was omitted because the domains were taken from a systematic literature review conducted prior to this study, which was an earlier objective of the broader research project.

Step 3: Emerging themes, statements, or comments related to a vision were organized based on the initial template outlined in Table 1.

**Table 1.** Initial template for catergorising data.

Subscale or domain	Prompt questions	Characteristics of comments or statements to include within the specified domain.
1. Education	What activities at school require children to have good vision?	1.1 Learning and Vision
		1.2 The Importance of Vision in Learning
		1.3 Learning difficulties resulting from impaired vision.
2. Distance vision	What role does vision play in children’s lives in general?	2.1 Distance vision activities and vision
		2.2 The importance of vision in distance vision activities
		2.3 Challenges with seeing far as a result of poor vision
3. Near vision	What role does vision play in children’s lives in general?	3.1 Near vision activities and vision
		3.2 The importance of vision on near vision activities
		3.3 Challenges with seeing near as a result of poor vision
	What role does vision play in children’s lives in general?	4.1 Movement and Vision
		4.2 The role vision plays in mobility and orientation
		4.3 Challenges with mobility and orientation if vision is impacted
4. Hobbies and Leisure	What types of fun things require children to have good eyesight?	5.1 Children’s hobbies and vision
		5.2 The Importance of Vision in fun activities children engage into
		5.3 Challenges with children’s hobbies or leisure if vision is impaired
5. Sports	What types of fun things require children to have good eyesight?	6.1 Sport and vision
		6.2 The role of vision in sporting activities
		6.3 How would performance in sports be impacted if vision is impacted?
6. Social interaction	Does a child's vision impact the way they form relationships?	7.1 Social engagement and vision
		7.2 The importance of vision in communication and relationships
		7.3 The impact of socializing as a result of poor vision
7. Visual Comfort	If a child's vision problem is identified, how would it impact their life and development?	8.1 Discomfort that children experience due to vision problems
		8.2 Impact of visual strains due to vision problems in children’s lives
8. Emotional	If a child's vision problem is identified, how would it impact their life and development?	9.1 Vision and Emotions
		9.2 Any relationship between vision and emotions, either positive or negative
9. Psychological	If a child's vision problem is identified, how would it impact their life and development?	10.1 Stress, anxiety, and depression concerning vision
10. Daily living activities/skills	What role does vision play in children’s lives in general?	11.1 Vision and the ability to perform daily tasks
		11.2 The impact of poor vision on the ability to perform daily tasks
11. Treatment	If a child's vision problem is identified, how would it impact their life and development?	12.1 Eyes and treatment
		12.2 The importance of managing eye problems
		12.3 Problems arising from the management of eye problems
12. Financial and family impact	If a child's vision problem is identified, how would it impact their life and development?	13.1 Management of eye problems and the cost
		13.2 The challenges faced by the family of a child with vision problems.

Step 4: The domains were defined as outlined in Table 1.

Step 5: Data was categorized according to the template in Table 1, and the template was updated based on the data during the data capture process.

Step 6: After completing the data analysis, the final template with data was created.

The statements were also classified as positive, negative, or neutral based on the emotional tone expressed. All statements were thoroughly reviewed and corrected before analysis. Access to the transcripts can be requested from the first author via email.

## Results

The experts consisted of four teachers focusing on learner support, two optometrists, two occupational therapists, and a nurse working at a paediatric hospital ward. All of these experts are currently working with children, and their years of experience range from 3 to 19 years. The majority of the participants in this group were female (n = 7), and all of them were African. A demographic profile of this group is detailed in Table 2.

**Table 2.** Demographic profile of the experts.

Participants code	PA1	PA2	PA3	PA4	PA5	PA6	PA7	PA8	PA9
Gender	Female	Female	Female	Female	Female	Female	Male	Male	Female
Occupation	Optometrist	Teacher	Occupational therapist	Teacher	Nurse	Optometrist	Teacher	Teacher	Occupational therapist
Years of experience	18	13	11	3	7	19	3	5	16

Seven FGDs were conducted with 49 children aged between 6-17 years, all of whom were African. The gender distribution was almost even, with 51% female and 49% male, with participants falling into the following age categories, 6-9 years (n = 22), 10-13 years (n = 15), and 14-17 years (n = 12). Of these, 34.7% had normal vision, 24.5% had refractive errors, and 40.8% had VI which could be categorized as ranging from moderate to blindness according to the WHO classification. ^16^ The children came from three schools located in the rural area of Limpopo province, Sekhukhune district. The demographics and visual profile of the children are outlined in Table 3.

**Table 3.** Demographic profile of children.

Participant code	Age	Gender	Dist-UVA R-eye in LogMAR	Dist-UVA L-eye in LogMAR	Near-UVA OU-in LogMAR	Cyclo-Auto-Ref R-eye	Cyclo-Auto-Ref L-eye	Dist-AVA R-eye	Dist-AVA L-eye	Near-AVA OU	Diagnosis	VA Classification
PB1	10	F	0.3	0.2	0	PL/-0.75X163	PL/-0.75X180	0	0	0	Astigmatism	RE
PB2	13	M	0.24	0	0	0.25/-0.50X175	0.25	0	0	0	Hyperopia Astigmatism	RE
PB3	15	F	0	0	0	PL	PL	0	0	0	NAD	Emmetropia
PB4	15	F	0.42	0.3	0	1.00/-1.25X15	1.00/-1.00X165	0	0	0	Hyperopia Astigmatism	RE
PB5	13	M	0	0.06	0	0.50/-0.50X90	0.25/-0.50X90	0	0	0	NAD	Emmetropia
PB6	13	F	0	0	0	PL	PL	0	0	0	NAD	Emmetropia
PB7	09	M	0.06	0.5	0	0.25/-0.25x180	-0.50/-0.50X180	0	0	0	Hyperopia, Astigmatism, Myopia	RE
PB8	07	M	0	0	0	PL	PL	0	0	0	NAD	Emmetropia
PB9	14	M	0.12	0.1	0	0.50/-0.50X73	0.50/-0.50X65	0	0	0	Hyperopia Astigmatism	RE
PB10	08	F	0	0	0	PL	PL	0	0	0	NAD	Emmetropia
PB11	07	M	0	0	0	PL	PL	0	0	0	NAD	Emmetropia
PB12	16	M	0.32	0.22	0	-0.75/-0.50X134	-0.75/-0.25X103	0	0	0	Myopia Astigmatism	RE
PB13	09	F	0	0	0	PL	PL	0	0	0	NAD	Emmetropia
PB14	11	F	0	0	0	PL	PL	0	0	0	NAD	Emmetropia
PB15	14	F	0	0	0	PL	PL	0	0	0	NAD	Emmetropia
PB16	14	F	0.62	0.52	0	-2.00/-0.25X138	-1.50/-0.50X118	0	0	0	Myopia Astigmatism	RE
PB17	15	M	0	0	0	PL	PL	0	0	0	NAD	Emmetropia
PB18	17	F	0	0	0	PL	PL	0	0	0	NAD	Emmetropia
PB19	12	M	0	0	0	PL	PL	0	0	0	NAD	Emmetropia
PB20	16	F	0	0	0	PL	PL	0	0	0	NAD	Emmetropia
PB21	12	M	0	0	0	PL	PL	0	0	0	NAD	Emmetropia
PB22	08	F	0.42	0.44	0	1.25/-1.75X03	1.00/-1.25X180	0	0	0	Hyperopia Astigmatism	RE
PB23	09	M	0	0	0	PL	PL	0	0	0	NAD	Emmetropia
PB24	11	F	0	0	0	PL	PL	0	0	0	NAD	Emmetropia
PB25	07	M	0.3	0.98	0	-1.00/-0.25x64	-0.5	0	0	0	Myopia Astigmatism	RE
PB26	06	M	0	0	0	PL	PL	0	0	0	NAD	Emmetropia
PB27	06	F	0.22	0.6	0	PL/-0.75X93	-0.25/-0.50X103	0	0	0	Myopia Astigmatism	RE
PB28	08	M	1.08	1.38	1	5.00/-7.25X102	7.00/-6.75X34	0.84	0.94	0.7	OCA	Moderate VI
PB29	08	M	1.3	1.18	0.1	3.75/-4.75x07	3.75/-3.75X12	0.56	0.52	0	OCA	Mild VI
PB30	07	F	0.7	0.7	0.7	3.00/-2.75X135	3.00/-2.25X45	0.3	0.26	0	OCA	Mild VI
PB31	06	F	2.04	2	1	Not Possible	Not Possible	2	2	1	ROP	Blind
PB32	06	F	NLP	NLP	NLP	NR	NR	NLP	NLP	NLP	Anophthalmia	Blind
PB33	06	M	1.4	1.4	0.9	-3.25/-4.25X78	-2.75/-5.00X65	0.72	0.72	0.3	Congenital Glaucoma)	Moderate VI
PB34	13	M	1.1	1.2	0.8	0.75/-3.50X115	1.00/-3.75X118	0.46	0.4	0.2	OCA	Mild VI
PB35	15	F	1.12	1.06	0.5	1.75/-5.25X17	2.50/-5.75X23	0.6	0.5	0	Nystagmus	Moderate VI
PB36	10	M	1.3	NLP	1	-6.25/-3.75X171	NR	1.3	NLP	1	Hypoplasia	Blind
PB37	11	M	1.32	2.02	1	-0.75/-1.00X93	-2.50/-1.75X06	1.32	2.02	1	Retinoschisis Secondary Optic atrophy	Severe VI
PB38	09	F	1.32	NLP	1.06	1.75/-5.50X169	0.50/-2.00X180	1.32	NLP	1.06	Congenital Glaucoma)	Blind
PB39	10	F	1.1	NLP	NR	9.25/-1.00X137	NR	0.6	NLP	NR	Aphakia Cataract	Severe VI
PB40	12	M	0.84	1.52	0.6	3.50/-4.75X1	6.75/-3.50X180	0.48	0.56	0.08	OCA	Mild VI
PB41	08	F	1.3	1.3	0.72	-2.75/-4.25X43	-3.25/-3.75X123	0.46	0.4	0	OCA	Mild VI
PB42	14	M	1.2	1	0.32	0.75/-8.25X03	0.50/-9.50X105	0.7	0.56	0.12	Keratoconus	Moderate VI
PB43	17	M	0.86	0.88	0.4	3.00/-4.75X18	3.00/-3.75X11	0.42	0.46	0	OCA	Mild VI
PB44	09	M	1.32	1.34	0.5	2.50/-4.25X100	2.50/-5.25X113	1.02	1.04	0.3	Keratoconus	Severe VI
PB45	08	F	1.1	1.1	0.3	4.75/-3.25X90	5.25/-3.75	0.28	0.36	0	OCA	RE
PB46	06	F	2	LP	NR	NR	NR	NR	NR	NR	Aniridia	Blind
PB47	10	F	0.46	0.46	0.7	1.25/-2.50X179	1.50/-2.25X111	0.14	0.14	0	OCA	RE
PB48	09	F	2	2	1	13.00/-0.75X90	12.25/-0.75X93	0.84	0.84	0.4	Aphakia	Moderate VI
PB49	11	M	1.1	1.2	0.6	PL/-6.25X15	1.00/-5.75X173	0.48	0.5	0.08	OCA	Mild VI

**F** = Female, M= Male, **Dist-UVA** = Distance Unaided Visual Acuity, **R**-eye= Right eye, **L**-eye= Left eye, **Near-UVA** = Near Unaided Visual Acuity, **OU** = Both eyes, **Cyclo-Auto-Ref** = Cycloplegic Auto Refraction, **Dist-AVA** = Distance Aided Visual Acuity, **Near-AVA** = Near Aided Visual Acuity, **NAD** = No Abnormality Detected, **NR** = No Record, **OCA** = Oculocutaneous Albinism, **ROP** = Retinopathy of prematurity, **RE** = refractive error, **VI** = Visual Impairment.

A total of 477 comments related to children’s vision were noted and categorized into the initially created template. Some domains were merged in the template and only left with 11 domains which are: ‘Education’, ‘mobility and orientation’, ‘Hobbies, leisure activities, and sports’, ‘social function’, ‘visual comfort’, ‘psychological and emotional’, ‘daily living activities/skills’, ‘self-esteem’, ‘family and financial impact’, and ‘treatment’ and, lastly sociocultural, which was generated specifically for this population of a rural school going children. The list generated is deposited in an online repository, accessible through this link: https://zenodo.org/records/13999156. Out of these, 299 (62.7%) statements were classified as negative, 92 (19.3%) were neutral, and 86 (18%) were positive. Furthermore, 286 (60%) of the comments emanated from the FGDs with children. These comments were further classified according to the template subscales or domains, and are outlined below. The domain “sociocultural” encompasses the factors that impact attitudes, values, and beliefs regarding VI and generated 13 statements.

## Summary of the generated domains, along with examples of statements assigned to each domain

### School and learning domain

One hundred items were generated under the education domain, these generated items illustrated the major role that vision plays in children’s learning and further highlighted the impact of vision problems on learning. Both children and experts agreed that VI affects aspects of learning, and the bearer of this condition goes through a lot. For instance, some statements noted under this domain included:

“ *I struggle to complete my school tasks on time due to poor vision*”. - PB22, an 8-year-old girl living with an uncorrected refractive error (URE).

“ *Most children with poor vision still struggle, even when seated at the front of the classroom*”. - PA8, teacher.

*“Most students with visual impairments resort to copying from their peers’ books in class due to difficulties in seeing the chalkboard”.* - PA2, teacher.

“ *I place my book very close when I read*”. - PB31, a six-year-old girl who is partially blind

### Daily living activities/skills domain

Under this domain a total of 47 items were generated. These items cover self-care tasks that children should be able to perform to care for themselves. These activities include personal hygiene, preparing meals and feeding, dressing oneself (such as tying shoes), and doing household chores. Vision problems can interfere with these daily activities. In this domain participants have highlighted the difficulties they experience with tasks like bathing, preparing meals, taking care of livestock, going to a store, and accurately using the right amount of something. Below are some related statements from the participants:

*“I cannot go to the tuck shop alone because I cannot see”.* - PB39, a 10-year-old girl who is severe VI.

*“My eyes help me to take livestock to the field for grazing”.* - PB23, a nine-year-old boy who is emmetropic.

*“I cannot bathe myself properly because I cannot see”.* - PB38, a nine-year-old girl who is blind.

*“Children with visual impairment struggle with preparing their meals”.* - PA7, teacher.

### Visual comfort domain

It is typical for children with visual impairments to suffer from headaches, eye strain, and fatigue. ^20^ The individual in this domain has described experiencing increased discomfort, itching, burning, strain, and headaches. Additionally, their symptoms are exacerbated in sunlight. A total of 25 items was generated under this domain, and below are some of the comments coming directly from some of the participants:

*“I often scratch my eyes because they are itching and burning”.* - PB17, a 15-year-old male who is emmetropic.

*“My eyes cause me lots of discomforts”.* - PB37, an 11-year-old boy living with severe VI) due to Retinoschisis.

*“Visually impaired children experience eye strains and headaches”.* - PA7, teacher.

*“I am very uncomfortable when there is too much sunlight”.* - PB35, a 15-year-old girl living with moderate VI.

### Mobility and orientation domain

Forty-seven items on this domain cover various aspects such as how vision helps children interpret and explore their surroundings, the challenges faced by visually impaired children in navigating new environments, and the potential risks and difficulties they encounter in this domain due to poor vision. Below are some of the statements directly from the participants:

“ *I need assistance when going to the pit toilet*”. - PB41, an eight-year-old girl, Mild VI.

“ *Children with visual impairment are prone to frequent falls and potential fractures due to walking challenges*”. - PA5, Nurse.

“ *I often trip on objects when moving within the classroom*”. - PB42, a 14-year-old boy, Moderate VI.

“ *Good vision is important for children to understand their environment*”. - PA6, Optometrist.

### Hobbies, leisure activities, and sports domain

Visual impairment has a noticeable effect on children’s hobbies, leisure activities, and participation in sports. Additionally, children with vision problems may find it challenging to watch TV, which can lead to feelings of loneliness and increased reserve behavior. ^4^ A hundred and one items were generated under this domain, and below are some of the statements stated by participants stated under this domain:

“ *Poor vision affects children’s ability to play, which affects their learning*”. -PA9, Occupational Therapist.

“ *I cannot play all the games I want to due to my poor eyesight*”. - PB38, a nine-year-old girl classified as blind.

“ *I get closer to the television (TV) so that I can see well*”. - PB43, a 17-year-old boy, Mild VI.

*“Visually impaired children often become introverted and spend most of their time at home”.* – PA1, Optometrist.

### Treatment domain

Most of the school-going children with visual impairment do not comply with wearing their optical corrections. ^21^ The reason might be issues like the stigma attached to the devices, broken spectacles, headaches, and or strains when they wear them. ^21^ In this domain participants also outlined that spectacles strain them, they do not like putting eye drops in their eyes. Children and expert participants indicated the stigma attached to wearing optical devices and the bullying that comes with wearing them. Twenty-two items were generated and below are how some participants commented under this domain:

*“It is challenging for visually impaired children to wear optical devices because of the stigma attached to them”.* - PA7, teacher.

*“Children with visual impairment get bullied at school when they wear spectacles”.* - PA5, nurse.

*“My glasses are straining my eyes, that’s why I do not like wearing them”.* - PB44, a seven-year-old boy who is severe VI.

*“My eye drops are not comfortable”.* - PB33, a six-year-old boy living with moderate VI.

### Families and financial impact domain

Visual impairment significantly affects the lives of individuals and their families, friends, and society as a whole. ^22^ This domain houses 20 items, which include statements that highlight the financial burden of caring for a visually impaired child, family misunderstanding of the condition, and the dependency that arises from always needing someone at home to care for a child with VI as indicated by statements of some of the participants below:

*“Caring for a visually impaired child comes with financial costs”.* - PA4, teacher.

*“The lives of parents of children living with visual impairment are always impacted, as they have to make arrangements for special care”.* - PA2, teacher.

*“Parents and caregivers of visually impaired children bear a heavier burden than the children themselves”.* - PA6, Optometrist.

*"My parents believe that I am seeking attention when I express my visual difficulties".* - PB4, a 15-year-old girl living with URE.

### Social functioning domain

Visual cues play a crucial role in social interaction as they are the primary means of identifying other people and provide important non-verbal social information through facial expressions and body language. ^23^ This domain has 41 items that highlight the challenges visually impaired children face in developing bonds with family and friends, being excluded from many activities due to poor vision, and spending a significant amount of time alone due to their condition. Some statements related to this domain included:

“ *Good vision aids in the development of bonds with family members, peers, and caregivers*”. - PA5, Nurse.

“ *Visually impaired children struggle to interpret the facial expressions of those they are communicating with*”. - PA9, Occupational Therapist.

“ *I spend most of my time at home due to poor eyesight*”. - PB36, a 10-year-old boy who is blind.

“ *No one wants to play with me because I cannot see well*”. - PB29, an eight-year-old boy living with mild VI.

### Emotional and psychological

The mental health of children with vision impairment may be adversely affected because they tend to participate in fewer physical activities, have lower academic achievement, and are more socially isolated. ^24^ This domain has produced 61 statements highlighting the impact of VI on children’s mental health, including social exclusion, increased dependency on others for assistance, reduced participation in physical activities, and the impact on the parents of visually impaired children as well. Below are some examples of generated statements from participants:

*“Children with poor vision feel lonely most of the time and they sometimes develop depression”.* - PA5, nurse.

*“Visually impaired children feel frustrated when choosing careers because they are restricted by their poor vision”.* - PA4, teacher.

*“I feel like I am bothering people when I ask them for scores during sporting events”.* - PB48, a nine-year-old girl living with moderate VI.

*"I often feel left out and isolated in social situations because of the glasses I wear".* - PB16, a 14-year-old female living with URE.

### Sociocultural

Thirteen items were generated in this domain which includes an examination of how cultural beliefs, such as attributing visual impairment to witchcraft or relying on traditional herbal medicine, can lead to delays in the diagnosis and proper management of visual impairment. Furthermore, the statements indicate that a child’s visual impairment may be influenced by their family’s poverty, leading to the child being treated differently. The statements also mention that parents may feel ashamed to admit that their visually impaired child needs to attend a special school. This is how some of these participants stated it:

*“Associating visual impairment with witchcraft delays the diagnosis and prognosis of visual impairment and worsens the condition”.* - PA5, nurse.

*“Some parents feel ashamed to admit that their visually impaired children need to attend special schools”.* - PA8, teacher.

*“Poverty also influences how visually impaired children are treated in their families and communities”.* - PA2, teacher.

*“The use of herbal medication delays the diagnosis and management of visual impairment”.* - PA4, teacher.

## Discussion

This study analysed data from FGDs with children and experts to explore the impact of VI on the lives of children. The experts who participated in this study had at least three years of experience working with children and encountered those with VI in their field of work. Age and gender were considered when selecting the child participants, as the impact of VI varies with these factors. ^25^ To ensure the representation of all types of children, 34.7% of the participants had no VI, 24.5% had a refractive error, and 40.8% had moderate to severe VI. The data was classified into ten domains adopted from a systematic review of literature on instruments to measure vision-related quality among children and adolescents. ^26^ The tenth domain (Sociocultural) was created based on the extra data generated for the participants in this study.

Poor vision has a significant impact on reading and learning, as 70-85% of school content and activities rely on vision. ^3, 27^ Individuals with VI encounter unique obstacles within the educational system, regardless of the severity of their impairment. ^28^ This study presents a compilation of statements concerning the impact of poor vision on children, especially those with VI. These visually impaired children, when attempting to read, tend to put books too close, frequently stop, lose track, and skip lines. ^29– 31^ Additionally, it was reported that children with early-onset VI may experience delayed motor, language, emotional, social, and cognitive development, leading to lower levels of educational achievement. ^32^ Moreover, children with impaired vision may experience decreased hand-eye coordination, which impacts their ability to perform tasks such as personal hygiene, preparing meals, dressing themselves, and doing household chores. ^33^ Participants further highlighted that vision problems can also interfere with daily activities, estimating the distance of oncoming cars, and seeing what is happening in the field during sporting events.

Children experiencing vision problems often exhibit behaviors such as rubbing their eyes or blinking frequently, avoiding reading, having short attention spans, experiencing frequent headaches, and covering one eye when focusing. ^34^ Even in this study, these individuals have described experiencing increased discomfort, itching, burning, strain, and headaches, which are exacerbated in sunlight.

Vision has been reported to play a crucial role in understanding the environment, developing awareness, gathering information about the relationships between objects, and carrying out various cognitive and motor activities. ^28^ In the study, it was found that children use vision to engage with others, explore the world around them, and rely on vision for activities like walking, avoiding obstacles, and being cautious in different settings. The study also highlights the need for assistance and the risk of falls and fractures faced by visually impaired children. In support of these issues of frequent falls and fractures associated with VI, several studies reported on that too. ^27, 35, 36^ For children to perform well, effective eye-hand coordination, clear vision, and good depth perception are crucial visual abilities. ^33^ When children experience vision problems, it not only impacts their ability to play sports but also hinders their overall learning experience. ^24^ Participants in this study also highlighted that poor vision impacts on children’s ability to play, consequently affecting their overall learning and development. Furthermore, individuals in this study have reported that VI limit’s children from reaching their full potential and securing good jobs, which ultimately affects the socioeconomic status of their families and the children themselves in the future. Such statements were also reported by studies on the impact of VI on the socioeconomic status of bearer. ^37– 39^

Visual impairment forces individuals to rely on non-visual cues during social interactions, leading to reduced social engagement and participation in enjoyable activities. ^40^ While verbal cues can convey a significant amount of information, this information is often not accessible to untrained listeners. ^23^ This study also highlighted that VI children struggle with social engagement and sometimes show inappropriate emotions to situations. Not surprisingly numerous studies suggest that VI and blindness are risk factors for anxiety and depression. ^21, 23, 40, 41^ This study highlights that children with vision problems experience a noticeable effect on their hobbies and leisure activities, and may find it challenging to watch TV, which can lead to feelings of loneliness and increasingly reserved behavior. ^4^

Children with VI often experience lower self-esteem due to the unique challenges they face compared to sighted children. ^42^ Self-esteem, which refers to one’s evaluation of oneself, is significantly affected in children with VI. ^43^ This study has shown that many of these children struggle with low confidence levels and feelings of worthlessness. In fact, the children themselves have expressed how profoundly low their confidence levels are.

Children with VI are more likely to live in deprivation, negatively impacting their emotional and mental well-being. ^21^ Even in this study, participants produced statements that highlighted the impact of VI on mental health. Several statements about the influence of culture, beliefs, and traditions when dealing with VI were grouped under the sociocultural domain. The sociocultural factors within a community have a profound influence on the emotions, attitudes, values, beliefs, and interactions of its members concerning VI. ^44^ This includes an examination of how cultural beliefs, such as attributing VI to witchcraft or relying on traditional herbal medicine, can lead to delays in the diagnosis and proper management of VI. It is important to note that the significant suffering caused by blinding eye diseases often goes unnoticed, especially because avoidable visual loss disproportionately impacts those living in rural and urban poverty. ^32^

Among the various domains discussed, the majority of the statements, comprising 21% (n=100), centered around hobbies, leisure, and sports. This finding is supported by a study conducted by Sacchetti *et al*. in 2007, which highlighted that children were primarily concerned about engaging in sports, visiting the pool, and socializing with friends. ^45^ The study further revealed that children’s proficiency in sports can be notably hampered if they lack adequate visual skills. The education domain emerged with the second-highest number of statements, which is not surprising given that this group is primarily involved in school-related activities. Additionally, the psychological and emotional domain also garnered significant attention, shedding light on the profound psychological impact of VI on the participants in the study. These findings are consistent with previous studies, indicating that children with VI often experience psychological effects that can significantly influence their overall quality of life. ^23, 45, 46^

## Conclusion

This study offers a comprehensive insight into the impact of VI on school-aged children and young people particularly those living in rural areas. The domain, sociocultural, shows the uniqueness of that population being studied and it is recommended that there be a tool to measure the impact of VI on rural school-going children.

## Ethics and consent

The research followed the principles of the Declaration of Helsinki regarding research involving humans, and ethical clearance was obtained from the Biomedical Research Ethical Committee of the University of KwaZulu-Natal (BREC/00003939/2022). The first approval was on the 15 ^th^ of November 2022, and we applied for the extension which was approved on the 15 ^th^ of November 2023. Written Consent and assent forms were provided and signed by experts, children, and their parents prior to data collection.

## Author’s contributions

T.S.S., R.H., and Z.N.X. conceived and planned the experiments. T.S.S. conducted the experiments. T.S.S., R.H., and Z.N.X. reviewed and analyzed the results. T.S.S. drafted the manuscript, while R.H. and Z.N.X. edited and made modifications to it. All authors have accepted the final version and approved it for submission to the journal.

## Disclaimer

All opinions expressed in this manuscript are solely those of the authors and stem from their professional research. The information presented does not necessarily align with the official policies or positions of the affiliated institute or the publisher. The authors are accountable for the results, findings, and content of this article.

## Data Availability

Zenodo: Statements from the Focus Group Discussions with Children and Experts, DOI: 10.5281/zenodo.13999155. ^47^ The project contains the following underlying data:
• Comments or Statements from FGDs All 27October2024.pdf Data are available under the terms of the Creative Commons Zero “No rights reserved” data waiver (CC0 1.0 Public domain dedication). The recording data for this study cannot be deposited in the public domain because it contains personal information that could easily be traced back to the participants. However, the data is available upon request. To obtain it, please send an email to the corresponding author, M.T.S.S. This study adhered to the Consolidated Criteria for Reporting Qualitative Studies (COREQ), which consists of 32 items for reporting guidelines. Zenodo: COREQ Checklist for the paper: Impact of vision problems on children’s daily activities: Insights from a focus group discussion, DOI: 10.5281/zenodo.14343032. ^48^ The project contains the following Reporting guidelines:
•
COREQ for FGD Paper by Magakwe tss et al.docx Data are available under the terms of the Creative Commons Zero “No rights reserved” data waiver (CC0 1.0 Public domain dedication).
