# Supplementary material for: Impact of vision problems on children’s daily activities: Insights from a focus group discussion
Source: F1000Res. 2025 Mar 18;13:1538. Originally published 2024 Dec 20. [Version 2] doi: 10.12688/f1000research.159464.2 (PMC11986411; doi:10.12688/f1000research.159464.2)
Supplement: Peer review history

# Reviewer report: Reviewer response for version 2

**DOI:** 10.5256/f1000research.179286.r374402

**Published:** 2025-04-10

**Authors:** Isabel Neto

**Affiliations:** 1 University of Lisbon, Lisboa, Portugal

It was very interesting to read this work, And it adds upon priori research as it as a multidisciplinary perspective including physicians. 

 It was really interesting to see VI children challenges apart from learning such as social cultural, emotional and psychologic, visual comfort domain (and how it affects their daily lives): 

 There are some improvements :

 - the coding process can be improved, showing the number of coders and the reliability of the coding process. 

 I thank the authors for this multidisciplinary  paper.

Is the work clearly and accurately presented and does it cite the current literature?

Yes

If applicable, is the statistical analysis and its interpretation appropriate?

Not applicable

Are all the source data underlying the results available to ensure full reproducibility?

Yes

Is the study design appropriate and is the work technically sound?

Yes

Are the conclusions drawn adequately supported by the results?

Yes

Are sufficient details of methods and analysis provided to allow replication by others?

Yes

Reviewer Expertise:

HCI and HRI for children with disabilities

I confirm that I have read this submission and believe that I have an appropriate level of expertise to confirm that it is of an acceptable scientific standard.

# Reviewer report: Reviewer response for version 1

**DOI:** 10.5256/f1000research.175189.r356442

**Published:** 2025-02-14

**Authors:** Lauren J Lieberman

**Affiliations:** 1 The College at Brockport, Brockport, NY, USA

Impact of vision problems on children’s daily activities: Insights from a focus group discussion

1. This study was completed in the past. Please revise this manuscript to align with past tense in English. As it is written it goes back and forth throughout with current, future and past tense.
2. Do not use the word handicapped. Use the word children with a disability
3. Pg 3 paragraph 1 in the introduction don’t say school-going just say school age
4. Pg. 3 Same paragraph Don’t say impairment say disability
5. Pg. 3 3 ^rd^ paragraph what is an FGD? Introduce this before you use the acronym
6. Pg. 3 under Focus Group-you said the professionals worked with children. Did they specifically work with children with VI? If so please say it here.
7. Pg. 4 you describe the questions you used. Who generated these questions? Was this list of questions validated? In other words, did any experts review the questions with your purpose statement to ensure they are valid and will result in effective results? For example, you ask about “good vision”. This is so subjective I would never use such a subjective term. If you did not validate these questions you need to add this issue to the limitations of this study. If only one person generated the questions there is certainly bias.
8. Pg. 4 this sentence “All participating children were proficient in English and did not have any impairments apart from vision” do not say impairments say disabilities.
9. What questions did you use for the focus group of the children? Were these questions validated? If not then add this issue to your limitations.
10. This study is not a qualitative study. It appears that you already had a template that the statements fit into. A qualitative study allows the data to be the results with NO preconceived outcome. This structure of the table literally puts the responses into a box and does not allow for the findings to emerge. You need to look up what qualitative research is and then revise this study.
11. Under results do not say “normal vision” just say vision. Also, if 34% had no VI then why were they in the study?
12. Pg. 13 you state “For children to perform well, effective eyehand coordination, clear vision, and good depth perception are crucial visual abilities.” This is not true. There are many blind sports such as goalball, blind tennis, and blind soccer to name a few that require no vision. You say this several times throughout the manuscript. You need to change this belief as it is just not true. These are international sports so they are available to these children.
13. Your discussion highlights several important points, such as isolation and loneliness, the need for sports, and more. These insights could have emerged with a more in-depth analysis of your data, which would have allowed for a richer exploration of these themes in your paper
14. I suggest you forgo the need to try to fit this data into an existing list of criteria. Instead use the data you found and truly develop themes and subthemes so you can share the reality of the lived experiences of the participants. As this study reads now, the research questions used are not validated and you used the voices of the experts and the children to try and force them into an existing instrument. This is not the intention of qualitative research.

Unless these researchers revise the manuscript to better reflect the voices of the children involved, its contribution to the literature may be limited. The effort is clearly present; now, it would be valuable to focus on listening to the participants and sharing their perspectives more fully.

Is the work clearly and accurately presented and does it cite the current literature?

No

If applicable, is the statistical analysis and its interpretation appropriate?

No

Are all the source data underlying the results available to ensure full reproducibility?

No

Is the study design appropriate and is the work technically sound?

Partly

Are the conclusions drawn adequately supported by the results?

No

Are sufficient details of methods and analysis provided to allow replication by others?

No

Reviewer Expertise:

NA

I confirm that I have read this submission and believe that I have an appropriate level of expertise to state that I do not consider it to be of an acceptable scientific standard, for reasons outlined above.

# Response

**Published:** 2025-03-11

**Authors:** Tshubelela Sello Simon Magakwe

**Affiliations:** Optometry, University of KwaZulu-Natal, Durban, KwaZulu-Natal, South Africa

Impact of vision problems on children’s daily activities: Insights from a focus group discussion

1. This study was completed in the past. Please revise this manuscript to align with past tense in English. As it is written it goes back and forth throughout with current, future and past tense.

- **Author’s reply:** The manuscript has been reviewed and revised accordingly wherever applicable.

 

1. Do not use the word handicapped. Use the word children with a disability

- **Author’s reply:** thank you for that comment, the word handicap was replaced with disability

 

1. Pg 3 paragraph 1 in the introduction, don’t say school-going just say school age

- **Author’s reply:** The revision was made to correct school-going to school age

 

1. Pg. 3 Same paragraph Don’t say impairment say disability

- **Author’s reply: **The revision was made to replace the word impairment with disability

 

1. Pg. 3 3 ^rd^ paragraph what is an FGD? Introduce this before you use the acronym

- **Author’s reply: **The FGDs acronym was defined as the focus group discussions at the beginning of paragraph 3.

 

1. Pg. 3 under Focus Group-you said the professionals worked with children. Did they specifically work with children with VI? If so please say it here.

- **Author’s reply:** To bring more information and clarification, the below sentence was added to the manuscript of the section of the FGDS:
- “ *A purposive sampling method was employed to recruit relevant, information-rich participants, that specifically work with children living with VI. The participants included one nurse, two occupational therapists, two optometrists, and four teachers from both mainstream and special schools. Among the experts, one occupational therapist worked at a school for visually impaired students, two optometrists were based at a low vision clinic specializing in children, and two teachers were also from the visually impaired school. The other professionals generally worked with children, and had interacted with various children, living with and without visual impairments*.”

 

1. Pg. 4 you describe the questions you used. Who generated these questions? Was this list of questions validated? In other words, did any experts review the questions with your purpose statement to ensure they are valid and will result in effective results? For example, you ask about “good vision”. This is so subjective I would never use such a subjective term. If you did not validate these questions you need to add this issue to the limitations of this study. If only one person generated the questions there is certainly bias.

- **Authors’s reply: **extra information were added for some clarity, and the limitations were  added to the manuscript with a statement below:
- “The prompt questions used in the focus group discussions were never validated, which may introduce some level of bias. However, these questions were developed based on existing literature prior to the study and reviewed by all three authors. They served primarily as guidelines and follow-up questions were employed to gather additional information from participants as needed, helping us to achieve saturation in our data collection.”

 

1. Pg. 4, this sentence “All participating children were proficient in English and did not have any impairments apart from vision” does not say impairments say disabilities.

- **Author’s reply:** The revision was made to revise the word impairments into disabilities.

 

1. What questions did you use for the focus group of the children? Were these questions validated? If not then add this issue to your limitations.

- **Author’s reply: **Those are the questions used by experts and extra information was added to bring more clarity. The limitations statement was provided the same as the one for query number 7

 

1. This study is not a qualitative study. It appears that you already had a template that the statements fit into. A qualitative study allows the data to be the results with NO preconceived outcome. This structure of the table literally puts the responses into a box and does not allow for the findings to emerge. You need to look up what qualitative research is and then revise this study.

- **Author’s reply:** Thank you for the input. This study was an objective in a larger project. This study used purposive sampling to identify information-rich participants who took part in FGDs to give responses that addressed the research question. Discussions were recorded and captured verbatim. As such, the study was classified as qualitative. Thematic analysis was used to identify themes and create a framework in an earlier objective of the study (https://doi.org/10.3390/ijerph21081009). The FGD objective that this manuscript addresses used that framework to inform its line of questioning and its data analysis.

 

1. Under results, do not say “normal vision”. Just say vision. Also, if 34% had no VI then why were they in the study?

- **Author’s reply:** Including normally sighted children in the study aimed to capture a broader range of activities that all children engage in during their daily lives. Focusing solely on visually impaired children would restrict the activities to those that only they could participate in. By including normally sighted children, we can understand the full spectrum of activities that children, regardless of their disabilities, are expected to be involved in.

 

1. Pg. 13 you state “For children to perform well, effective eyehand coordination, clear vision, and good depth perception are crucial visual abilities.” This is not true. There are many blind sports such as goalball, blind tennis, and blind soccer to name a few that require no vision. You say this several times throughout the manuscript. You need to change this belief as it is just not true. These are international sports so they are available to these children.

- **Author’s reply: **This is well noted. All relevant sentences have been rephrased to indicate that eye hand coordination, clear vision and good depth perception are advantageous visual abilities, particularly for participation in sporting activities.

 

1. Your discussion highlights several important points, such as isolation and loneliness, the need for sports, and more. These insights could have emerged with a more in-depth analysis of your data, which would have allowed for a richer exploration of these themes in your paper

- **Author’s reply:** This is well noted, and will be taken into consideration for future manuscripts.

 

1. I suggest you forgo the need to try to fit this data into an existing list of criteria. Instead use the data you found and truly develop themes and subthemes so you can share the reality of the lived experiences of the participants. As this study reads now, the research questions used are not validated and you used the voices of the experts and the children to try and force them into an existing instrument. This is not the intention of qualitative research.

- **Author’s reply:** The reason for the predefined themes was that this manuscript is linked to a larger study, which aimed to develop a visual function quality of life instrument for rural school-aged children living with visual impairment. The data presented in this paper was focused on that objective. However, we approached this focus group discussion (FGD) with an open mind, which is why we were able to include a sociocultural theme or domain that we had not anticipated at the start of the data collection.

 

1. Unless these researchers revise the manuscript to better reflect the voices of the children involved, its contribution to the literature may be limited. The effort is clearly present; now, it would be valuable to focus on listening to the participants and sharing their perspectives more fully.

 

- **Author’s reply:** I can confirm that this study successfully gathered all the necessary information from the participants. Furthermore, the significance of the findings presented in this manuscript lies in the fact that we examined a population that has not been previously studied in the development of existing quality of life tools.

# Reviewer report: Reviewer response for version 1

**DOI:** 10.5256/f1000research.175189.r358837

**Published:** 2025-02-10

**Authors:** Asha Latha Mettla

**Affiliations:** 1 L V Prasad Eye Institute, Hyderabad, Telangana, India

Summary: The study is an addition of evidence to the existing literature on the importance of assessment of the quality of life of Children with visual impairment. This is a qualitative study conducted in the form of focus group discussions(FGDs') and experts and children were part of the discussions. Purposive sampling is used and children mapped to different levels of visual impairment were included into FGDs'. Template analysis is adapted to analyse the results of the FGDs'. The responses were categorised based on the codes available in the existing literature and the statements were further mapped based on the response type as neutral, positive and negative ones. The impact of visual impairment on the lives of children at different functional aspects  like motor, language, social, cognitive aspects and the impact on academic performance is discussed.

 Details related to responses given as "Partly::

 Methods: The categorisation of statements to different  domains/subscale and the classification of statements based one emotional tone is mentioned under the steps  followed. However, additional details on how this is ensured among the two individuals involved in allocating the responses to different domains may be required. It may give information about ensuring standardisation among the people and also will tell on how the disagreements between the coders is resolved while assigning the domains and assigning the comments to positive, negative or neutral. Parents, an important part of children's daily life are not included into the group discussions. A few lines mentioning the rationale for not including parents may be useful.

 Results: If possible, tables that show the results of the focus group discussions may be given for the children and the experts, separately. It helps to give a clear understanding on the outcomes of the discussions based on the categories versus the responses. This may be a basis to develop the tool for children to measure the impact, as given in conclusion.

 Discussion: The importance of the results and how it helps in giving a basis for the requirement of a separate tool may be brought into the discussion. It will strengthen the idea of developing the items of the questionnaire.

Is the work clearly and accurately presented and does it cite the current literature?

Yes

If applicable, is the statistical analysis and its interpretation appropriate?

I cannot comment. A qualified statistician is required.

Are all the source data underlying the results available to ensure full reproducibility?

Partly

Is the study design appropriate and is the work technically sound?

Yes

Are the conclusions drawn adequately supported by the results?

Yes

Are sufficient details of methods and analysis provided to allow replication by others?

Partly

Reviewer Expertise:

Health economics, Project management and Children Eye Care in Public Health.

I confirm that I have read this submission and believe that I have an appropriate level of expertise to confirm that it is of an acceptable scientific standard.

# Response

**Published:** 2025-03-11

**Authors:** Tshubelela Sello Simon Magakwe

**Affiliations:** Optometry, University of KwaZulu-Natal, Durban, KwaZulu-Natal, South Africa

**Reviewer: Asha Latha Mettla**

 Summary: The study is an addition of evidence to the existing literature on the importance of assessment of the quality of life of Children with visual impairment. This is a qualitative study conducted in the form of focus group discussions(FGDs') and experts and children were part of the discussions. Purposive sampling is used and children mapped to different levels of visual impairment were included into FGDs'. Template analysis is adapted to analyse the results of the FGDs'. The responses were categorised based on the codes available in the existing literature and the statements were further mapped based on the response type as neutral, positive and negative ones. The impact of visual impairment on the lives of children at different functional aspects  like motor, language, social, cognitive aspects and the impact on academic performance is discussed.

 Details related to responses given as "Partly::

  

1. Methods: The categorisation of statements to different  domains/subscale and the classification of statements based one emotional tone is mentioned under the steps  followed. However, additional details on how this is ensured among the two individuals involved in allocating the responses to different domains may be required. It may give information about ensuring standardisation among the people and also will tell on how the disagreements between the coders is resolved while assigning the domains and assigning the comments to positive, negative or neutral.

 

- Author's reply: Some information was added to bring clarity to the query above within the body of the manuscript:

“PI and SS initially chart and distribute data independently by domain, and later meet to discuss and finalise the charted and distributed data”

  

1. Parents, an important part of children's daily life are not included into the group discussions. A few lines mentioning the rationale for not including parents may be useful.

- Author’s reply: Thank you for the comment, this imported point was noted and put as a limitation as below statement:

“Parents play a very important role in their children's daily lives, so not including them in this study is recognized as a limitation. However, teachers spend most of their time with school-aged children, and having four of them involved in the study helped in collecting the enriched data that was needed.”

  

1. Results: If possible, tables that show the results of the focus group discussions may be given for the children and the experts, separately. It helps to give a clear understanding on the outcomes of the discussions based on the categories versus the responses. This may be a basis to develop the tool for children to measure the impact, as given in conclusion.

- Author’s reply: A table was created during the preparation of this manuscript (see below), but it was removed to shorten the document. The information originally included in the table is already presented in the results section under the heading "Summary of the Generated Domains," along with examples of statements assigned to each domain.

 

1. Discussion: The importance of the results and how it helps in giving a basis for the requirement of a separate tool may be brought into the discussion. It will strengthen the idea of developing the items of the questionnaire.

- Author’s reply: The below statement was added to motivate the development of VRQoL tool.
- “This study highlights the need to develop a tool specifically designed for rural South African children, even though other tools already exist. Additionally, the findings of this study contribute to the existing literature by emphasizing the importance of incorporating the sociocultural domain into quality of life (QoL) tools”.
